# Cloud Computing for Infectious Disease Surveillance and Control: Development and Evaluation of a Hospital Automated Laboratory Reporting System

**DOI:** 10.2196/10886

**Published:** 2018-08-08

**Authors:** Mei-Hua Wang, Han-Kun Chen, Min-Huei Hsu, Hui-Chi Wang, Yu-Ting Yeh

**Affiliations:** ^1^ Graduate Institute of Biomedical Informatics College of Medical Science and Technology Taipei Medical University Taipei Taiwan; ^2^ Department of General Surgery Chi-Mei Medical Center Tainan Taiwan; ^3^ Information Technology Office Shuang Ho Hospital Taipei Medical University New Taipei City Taiwan

**Keywords:** laboratory autoreporting system, HALR, electronic medical records

## Abstract

**Background:**

Outbreaks of several serious infectious diseases have occurred in recent years. In response, to mitigate public health risks, countries worldwide have dedicated efforts to establish an information system for effective disease monitoring, risk assessment, and early warning management for international disease outbreaks. A cloud computing framework can effectively provide the required hardware resources and information access and exchange to conveniently connect information related to infectious diseases and develop a cross-system surveillance and control system for infectious diseases.

**Objective:**

The objective of our study was to develop a Hospital Automated Laboratory Reporting (HALR) system based on such a framework and evaluate its effectiveness.

**Methods:**

We collected data for 6 months and analyzed the cases reported within this period by the HALR and the Web-based Notifiable Disease Reporting (WebNDR) systems. Furthermore, system evaluation indicators were gathered, including those evaluating sensitivity and specificity.

**Results:**

The HALR system reported 15 pathogens and 5174 cases, and the WebNDR system reported 34 cases. In a comparison of the two systems, sensitivity was 100% and specificity varied according to the reported pathogens. In particular, the specificity for *Streptococcus pneumoniae*, *Mycobacterium tuberculosis* complex, and hepatitis C virus were 99.8%, 96.6%, and 97.4%, respectively. However, the specificity for influenza virus and hepatitis B virus were only 79.9% and 47.1%, respectively. After the reported data were integrated with patients’ diagnostic results in their electronic medical records (EMRs), the specificity for influenza virus and hepatitis B virus increased to 89.2% and 99.1%, respectively.

**Conclusions:**

The HALR system can provide early reporting of specified pathogens according to test results, allowing for early detection of outbreaks and providing trends in infectious disease data. The results of this study show that the sensitivity and specificity of early disease detection can be increased by integrating the reported data in the HALR system with the cases’ clinical information (eg, diagnostic results) in EMRs, thereby enhancing the control and prevention of infectious diseases.

## Introduction

Electronic laboratory reporting (ELR) generally refers to the automated transmission of reportable laboratory findings from public health services, hospitals, and other laboratories to local or state public health agencies. Many communicable diseases currently under nationwide surveillance can be identified and confirmed by laboratory test results. Thus, ELR has become a critical part of the disease surveillance process. Previous studies have shown that ELR improves the timeliness, accuracy, and completeness of reported laboratory data, which, in turn, improves the effectiveness and efficiency of public health responses to outbreaks and cases of notifiable conditions [1-4]. It has been included as a meaningful use objective by the US Centers for Medicare and Medicaid Services’ Electronic Health Records Incentive programs [5,6]. However, ELR often lacks the clinical information needed to satisfy a case definition, such as disease signs, symptoms, and diagnoses. Moreover, ELR is usually nonspecific, particularly in cases (such as acute hepatitis B) where diagnoses require integration of laboratory tests and clinical information [7-10]. The lack of specificity in ELR increases the workload for health departments compelled to investigate suggestive but nonspecific lab results [11].

An electronic medical record (EMR) is a systematized collection of computerized patient and population health information. An EMR supports secure, real-time, point-of-care, patient-centric information availability and is a resource for clinical care as well as research and education. Recently, EMR systems have become an increasingly pervasive technology in health care settings [12-14]. Several studies have shown that EMR systems can accelerate clinical information flow, facilitate health care data integration, and improve the efficiency and quality of medical services [15-17]. With the growing adoption of EMR systems in recent years, increasingly sophisticated data have become available in EMRs to support infection surveillance, prevention, and control [18-20]. Using EMR data for the detection and reporting of infectious diseases also has the potential to improve public health monitoring and reporting [21]. However, current EMR systems are primarily built to serve clinical practice and are not structured for public health use. Laboratory test data transmitted to EMR systems might not be as complete and as timely as data received directly from a laboratory information system (LIS) [22].

In Taiwan, as required by law [23], clinical laboratory units operated by hospitals, health agencies, and research institutes must report the outcomes of tests for pathogens, mostly bacteria and viruses, that meet the notification conditions of Taiwan’s Centers of Disease Control (TCDC) in order to enable epidemiological surveillance and advanced alerting of communicable diseases. Thus, the TCDC has developed a Web-based Notifiable Disease Reporting (WebNDR) system to strengthen infectious disease control and surveillance. The system requires surveillance professionals to enter detailed information of notifiable diseases and laboratory tests manually if the diseases meet the TCDC’s reporting definitions. Unfortunately, these reporting operations are time consuming and error prone [24]. This is also the case for unannounced or delayed notifications [25].

In order to reduce the workload of surveillance professionals, and address the lack of specificity of ELR, the TCDC launched a pilot project, called Automated Laboratory Reporting (ALR), in 2014 [26]. The ALR system enables a hospital to automatically transmit reportable cases to the TCDC when they meet TCDC’s notifiable conditions. Reportable cases include not only detailed laboratory test data but also relevant EMR data. Currently, hospitals can join the project on a voluntary basis. The TCDC offers incentives to help develop a counterpart to the TCDC’s ALR system in hospital settings. Although including EMR data into laboratory-reportable cases can improve the specificity of ELR, few studies have evaluated such systems. In this study, we developed a counterpart to the TCDC’s ALR system for a hospital setting and evaluated its effectiveness in terms of sensitivity and specificity compared with the existing WebNDR system.

## Methods

### Settings

In this study, we implemented a counterpart of the TCDC’s ALR system in a teaching affiliate hospital with about 1000 beds starting in August 2014 in Taiwan. The hospital has an LIS to manage laboratory orders and results, a computerized physician order entry (CPOE) system to support clinicians in their daily clinical practice including entering test orders and reviewing test results, and other EMR systems. It can also access the WebNDR system to report notifiable diseases manually.

### The Framework of Automated Laboratory Reporting System in a Hospital Setting

**Figure 1:**
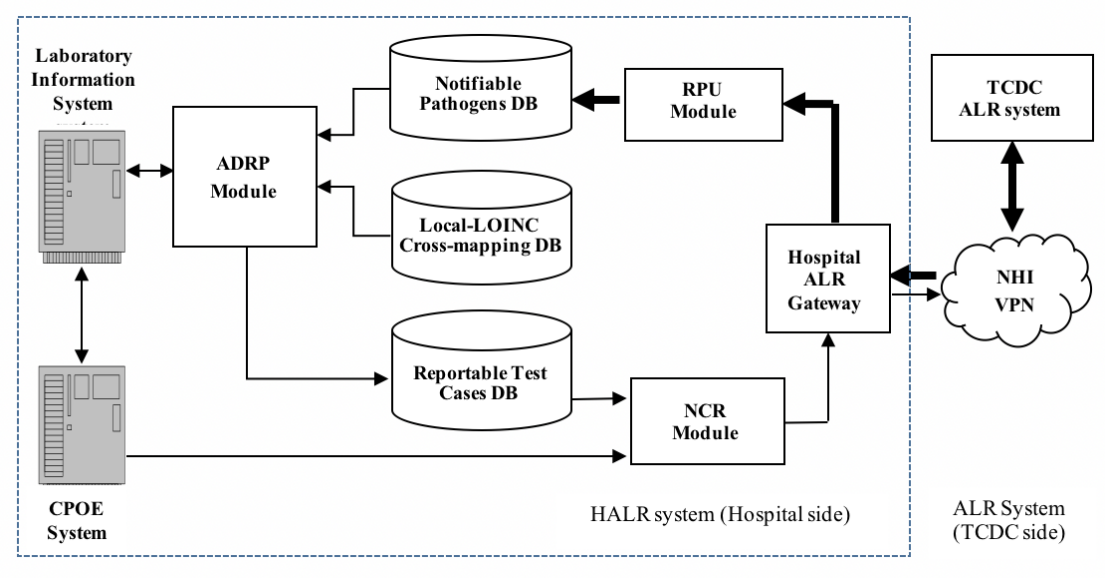
The framework of a Hospital Automated Laboratory Reporting (HALR) System. (The thick “-” corresponds to the out-of-hospital system; the thin “-” corresponds to the in-hospital system). ADRP: Automated Detection of Reportable Pathogens, ALR: Automated Laboratory Reporting, CPOE: computerized physician order entry, DB: database, LOINC: Logical Observation Identifiers Names and Codes, NCR: Notifiable Case Reporting, NHI: National Health Insurance, RPU: Reportable Pathogens Update, TCDC: Taiwan’s Centers of Disease Control, VPN: virtual private network.

Based on the framework of the TCDC’s ALR system, this study documents the implementation in a hospital setting, termed the Hospital Automated Laboratory Reporting (HALR) system (Figure 1). The HALR system consists of four major modular components: HALR Gateway, Reportable Pathogens Update (RPU), Automated Detection of Reportable Pathogens (ADRP), and Notifiable Case Reporting (NCR). The HALR Gateway is responsible for downloading the latest notifiable disease pathogens from the TCDC’s ALR system and uploading reportable cases to the TCDC ALR system for further processing. Using the RPU module, the downloaded pathogens are then used to update the Notifiable Pathogen Database (NPDB) in the HALR system. This belongs to the out-of-hospital system data and is indicated by a thick line.

When the LIS releases a pathogen test result, the ADRP module checks the pathogen against the NPDB. LIS pathogen test results include patient ID, name, gender, test item, specimen type, and pathogen report content. If the pathogen can be found in the NPDB and its test result is positive, the test case is labeled as reportable. The patient’s information is then included with the pathogen test result to form a reportable case. The laboratory test is encoded with a hospital code; however, the TCDC requires a laboratory test with a Logical Observation Identifiers Names and Codes (LOINC) code.

Thus, the ADRP module can translate the hospital code of a pathogen test into its corresponding LOINC code by referencing a local LOINC cross-mapping database. The reportable test cases detected by the ADRP module are then stored into the Reportable Test Case Database (RTCDB).

Finally, the NCR module retrieves a reportable test case from the RTCDB and links the test data with the patient’s clinical information, such as disease name, diagnosis code, and the date when the condition was diagnosed, which are stored in the CPOE system. Then, the linked data are compiled into a laboratory-reportable case in a digital format such as XML that is defined by the TCDC. Subsequently, the HALR Gateway sends the laboratory-reportable case to the TCDC ALR system; this belongs to the in-hospital system data and is indicated by a thin line.

### Mapping Between Pathogens and Diseases

When the LIS confirms a pathogen test result that meets a laboratory reporting condition, the pathogen test result and other information, such as order number, local code, specimen type, and time of result, will be automatically written into the RTCDB.

After retrieving new reporting data, the program converts the laboratory test items to LOINC codes and retrieves other patient information required by the TCDC from different hospital information systems and then submits the combined data to the hospital reporting module within the TCDC gateway in the required format.

Since the WebNDR system is dedicated to reporting communicable diseases, the five disease categories reported by this system are used as a basis of evaluating the HALR system. The TCDC provides a table for mapping between pathogens and their related diseases (Table 1).

### System Evaluation

After the HALR system was implemented in a regional hospital in Taipei, we collected reported cases from the HALR and WebNDR systems for 6 months, from December 2014 to May 2015. Since the reported cases from the WebNDR system were confirmed by infectious control professionals, they served as a gold standard for evaluating the HALR system in this study. Thus, the sensitivity and specificity of the reported cases by the HALR system could be evaluated according to the following definitions:

Sensitivity (true positive rate): If a patient has a notifiable disease reported by the WebNDR system, the sensitivity is the probability that the HALR system would report the case. The numerator is the number of cases identified as positive and reported by both the HALR and WebNDR systems, whereas denominator is the total number of cases reported by the WebNDR system (# of reported cases by both the HALR and WebNDR systems)/(# of cases reported by the WebNDR system).

Specificity (true negative rate): If a patient does not have a notifiable disease and is not reported by the WebNDR system, the specificity is the probability that the HALR system would not report the case. The numerator is the number of cases in whom test results were identified as negative and were not reported by the WebNDR system, whereas the denominator is the total number of cases not reported by the WebNDR system (# of not reported cases by neither the HALR nor WebNDR system)/(# of cases not reported by the WebNDR).

**Table 1:** Mapping between pathogens and diseases (based on the Web-based Notifiable Disease Reporting system).

Pathogen	ICD-9-CM^a^	Disease
*Streptococcus pneumoniae*	481, 482, 485, 486, 038, 041, 320	Invasive pneumococcal disease
*Mycobacterium tuberculosis* complex	010-018	Tuberculosis
Influenza virus	487	Severe complicated influenza case
Hepatitis B virus	070.20,070.21,070.30, 070.31	Acute hepatitis B
Hepatitis C virus	070.41	Acute hepatitis C

^a^ICD-9-CM: The International Classification of Diseases, Ninth Revision, Clinical Modification.

**Table 2:** The predictive specificity and sensitivity values of tests evaluated for the Hospital Automated Laboratory Reporting (HALR) system (pathogen test result) compared with those for the Web-based Notifiable Disease Reporting (WebNDR) system (reported result).

Method	Reported by the WebNDR system	Not reported by the WebNDR system	Total
Reported (positive) by the HALR system	TP^a^	(FP^b^)	TP+(FP)
Not reported (negative) by the HALR system	(FN^c^)	TN^d^	(FN)+TN
Total	TP+(FN)	(FP)+TN	—

^a^TP: true positive.

^b^FP: false positive.

^c^FN: false negative.

^d^TN: true negative.

The cases reported by the WebNDR system were patients with notifiable diseases, whereas those reported by the HALR system were patients whose pathogen test results were positive. During his or her hospital stay, a patient with the same pathogen reported more than once (ie, a repeatedly reported case) was excluded.

Furthermore, since a pathogen may cause more than one disease, the HALR system includes clinical diagnosis information such as that from the International Classification of Diseases, Ninth Revision, Clinical Modification (ICD-9-CM). Thus, the test results were combined with ICD-9-CM data to determine whether the HALR system performance could be improved.

- Sensitivity (with ICD): The numerator is the number of cases identified as a notifiable disease with a positive test result, which were automatically reported by the HALR and WebNDR systems. The denominator is the total number of cases reported by the WebNDR system.
- Specificity (with ICD): The numerator is the number of cases identified as negative and, therefore, not reported by either the HALR or WebNDR system. The denominator is the total number of cases not reported by the WebNDR system (Table 2).

## Results

### Pathogens Analysis

Based on the TCDC’s reportable pathogens and reporting criteria, 15 pathogens were reported to the TCDC ALR system (Table 3). Among these, 5174 patients had positive test results and were reported by the HALR system.

Simultaneously, 34 cases in five disease categories were reported by the WebNDR system (Table 4). The number of reported cases from different disease categories was pulmonary tuberculosis (25), complications from severe influenza (3) and acute viral hepatitis C (3), invasive *Streptococcus pneumoniae* infection (2), and acute viral hepatitis B (1).

### Sensitivity and Specificity Analysis

Table 5 shows the sensitivity and specificity analysis for the HALR system. In this study, the sensitivity for the HALR system reached 100%, but the specificity varied according to the pathogens. The specificity for *S. pneumoniae*, *Mycobacterium tuberculosis* complex, and hepatitis C virus were 99.8%, 96.6%, and 97.4%, respectively. In addition, the specificity for influenza and hepatitis B virus were only 79.9% and 47.1%, respectively.

Because the HALR system collects a reported case that includes not only laboratory test results but also clinical information (such as ICD-9-CM) from the TCDC ALR system, only cases with associated laboratory positive test results and an associated diagnosis code that meets the TCDC’s notifiable disease definition are reported to the TCDC ALR system (Table 6). The sensitivity and specificity of cases reported by the HALR system can be recalculated (Table 6). The sensitivity performance remains unchanged, but the specificity is greatly improved, in particular for influenza (89.2%) and hepatitis B virus (99.1%). Thus, the inclusion of clinical information in the reported data can improve the specificity performance.

**Table 3:** The analysis of laboratory test results during the study period.

Name of the detected pathogen		Total number of subjects (N=57,511), N (%)		Pathogen test results, n (%)			
				Negative (n=52,337)		Positive (n=5174)	
**Notifiable diseases (law requirements)**							
	*Salmonella* spp		10,201 (17.8)		10,173 (19.4)		28 (0.5)
	*Streptococcus pneumoniae*		9970 (17.3)		9946 (19.0)		24 (0.5)
	*Mycobacterium tuberculosis* complex		1622 (2.8)		1542 (3.0)		80 (1.5)
	Influenza virus		1980 (3.4)		1579 (3.0)		401 (7.8)
	Enterovirus		N/A^a^		N/A		N/A
	Hepatitis B virus		7917 (13.8)		3725 (7.1)		4192 (81)
	Hepatitis C virus		5124 (8.9)		4986 (9.5)		138 (2.7)
**Nonnotifiable diseases**							
	*Streptococcus agalactiae*, GBS^b^		10,576 (18.4)		10,356 (19.8)		220 (4.2)
	*Streptococcus pyogenes*		9958 (17.3)		9898 (18.9)		60 (1.2)
	Parainfluenza virus		N/A		N/A		N/A
	Respiratory syncytial virus		90 (0.2)		80 (0.2)		10 (0.2)
	Rotavirus		73 (0.1)		52 (0.1)		21 (0.4)
	*Yersinia enterocolitica*		N/A		N/A		N/A
	*Campylobacter* spp		N/A		N/A		N/A
	*Listeria monocytogenes*		N/A		N/A		N/A

^a^N/A: not applicable.

^b^GBS: Group B Streptococcus.

**Table 4:** The analysis of the Web-based Notifiable Disease Reporting system during the study period.

Name of the detected pathogen	Total number of reported cases, n (%)
*Streptococcus pneumoniae*	2 (6)
*Mycobacterium tuberculosis* complex	25 (73)
Influenza virus	3 (9)
Hepatitis B virus	1 (3)
Hepatitis C virus	3 (9)

**Table 5:** The sensitivity and specificity of the Hospital Automated Laboratory Reporting (HALR) system.

Pathogen name and HALR system test result		WebNDR^a^ system		Total	Sensitivity or specificity, %
		Reported, n (%)	Not reported, n (%)		
***Streptococcus pneumoniae***					
	Positive (reported)	2 (100)	22 (0.2)	24	100^b^
	Negative (not reported)	0 (0)	9946 (99.8)	9946	99.8^c^
	Total	2 (100)	9968 (100)	9970	—
***Mycobacterium tuberculosis* complex**					
	Positive (reported)	25 (100)	55 (3.4)	80	100^b^
	Negative (not reported)	0 (0)	1542 (96.6)	1542	96.6^c^
	Total	25 (100)	1597 (100)	1622	—
**Influenza virus**					
	Positive (reported)	3 (100)	398 (20.1)	401	100^b^
	Negative (not reported)	0 (0)	1579 (79.9)	1579	79.9^c^
	Total	3 (100)	1977 (100)	1980	—
**Hepatitis B virus**					
	Positive (reported)	1 (100)	4191 (52.9)	4192	100^b^
	Negative (not reported)	0 (0)	3725 (47.1)	3725	47.1^c^
	Total	1 (100)	7916 (100)	7917	—
**Hepatitis C virus**					
	Positive (reported)	3 (100)	135 (2.6)	138	100^b^
	Negative (not reported)	0 (0)	4986 (97.4)	4986	97.4^c^
	Total	3 (100)	5121 (100)	5124	—

^a^WebNDR: Web-based Notifiable Disease Reporting.

^b^Refers to sensitivity.

^c^Refers to specificity.

**Table 6:** The analysis of sensitivity and specificity between the Hospital Automated Laboratory Reporting (HALR) and Web-based Notifiable Disease Reporting (WebNDR) systems with the International Classification of Diseases, Ninth Revision, Clinical Modification (ICD-9-CM) codes.

Pathogens name (ICD-9-CM code) and result		WebNDR system		Total	Sensitivity or specificity (%)
		Reported, n (%)	Not reported, n (%)		
***Streptococcus pneumoniae* (ICD-9-CM: 485, 486)**					
	Positive (reported)	2 (100)	12 (0.1)	14	100^a^
	Negative (not reported)	0 (0)	9956 (99.9)	9956	99.9^b^
	Total	2 (100)	9968 (100)	9970	—
***Mycobacterium tuberculosis* complex (ICD-9-CM: 011.00, 011.90)**					
	Positive (reported)	25 (100)	12 (0.8)	37	100^a^
	Negative (not reported)	0 (0)	1585 (99.2)	1585	99.2^b^
	Total	25 (100)	1597 (100)	1622	—
**Influenza virus (ICD-9-CM: 487.1)**					
	Positive (reported)	3 (100)	214 (10.8)	217	100^a^
	Negative (not reported)	0 (0)	1763 (89.2)	1763	89.2^b^
	Total	3 (100)	1977 (100)	1980	—
**Hepatitis B virus (ICD-9-CM: 070.30)**					
	Positive (reported)	1 (100)	74 (0.9)	75	100^a^
	Negative (not reported)	0 (0)	7842 (99.1)	7842	99.1^b^
	Total	1 (100)	7916 (100)	7917	—
**Hepatitis C virus (ICD-9-CM: 070.41, 070.51)**					
	Positive (reported)	3 (100)	9 (0.2)	12	100^a^
	Negative (not reported)	0 (0)	5112 (99.8)	5112	99.8^b^
	Total	3 (100)	5121 (100)	5124	—

^a^Refers to sensitivity.

^b^Refers to specificity.

## Discussion

### Principal Findings

In this study, we developed an HALR system for the automatic reporting of pathogen test results and clinical information. Once laboratory test results are released, the HALR system can automatically detect pathogens that meet notifiable conditions as defined by the TCDC and report the cases to the TCDC ALR system. Since the patients’ laboratory test results are usually released to their physicians far in advance of the physicians’ final diagnoses of any notifiable diseases, the HALR system can improve the timeliness for notifiable disease surveillance and control. Moreover, this system’s effectiveness is also improved as long as the doctor has included a working diagnosis.

As indicated in previous studies [7-10], an ELR is usually nonspecific. The analysis reported in this study showed that if reported data only included the results of laboratory tests, the specificity of the reported cases by the HALR system for some diseases would be quite low. This could lead to an increase in reported cases as well as increase in workload related to the investigation of suggestive cases. However, if reported data were slightly augmented with clinical information, such as the clinical diagnosis code, the specificity of the reported cases could be greatly improved. The primary reason is that a given pathogen may cause several different diseases, which are not notifiable. For example, a hepatitis B virus infection may lead to acute or chronic hepatitis, but only acute hepatitis B (with ICD-9-CM diagnosis code 070.30) must be reported. Similarly, influenza viruses may cause acute respiratory infections, from mild to severe, but only cases with severe complications from influenza (ICD-9-CM 487.1) must be reported. However, because influenza is caused by a variety of pathogens and easily causes pandemics, the National Respiratory and Enteric Virus Surveillance System of the World Health Organization provides influenza-like illnesses with clear definitions and clinical symptom identification standards, which must be updated and revised according to emerging viruses and changes in patients’ disease features. The HALR system reports not only laboratory test results but also relevant clinical information and, thus, can greatly improve its performance.

In response to emerging infectious diseases, the HALR system detects reportable cases based on the NPDB. This design allows the TCDC to add, delete, or update the definitions of reportable pathogens as new emerging threats are identified. Hence, as this system can routinely download and update the NPDB, it can quickly respond to altered requirements.

The improvement of the sensitivity and specificity of reportable cases depends not only on timely and accurate laboratory test results but also on the availability of clinical information required to identify cases. This study simultaneously examined reporting data on the basis of test results and clinical evidence in order to enable data sharing between information systems. Through double verification, with this approach, hospitals can immediately confirm patients’ conditions and submit reports when required. Thus, the sensitivity and specificity for early disease detection can be improved. The findings of this study can serve as a reference for disease prevention measures in clinical care.

### Limitations

The following are some limitations of this system. First, the system will be limited to tests accepted by patients in the hospital. This system can only automatically link information when required clinical data are complete. Second, the notification condition of the test and the positive definition of the pathogen remain important factors affecting the accuracy of the content of the notification.

### Conclusions and Future Directions

In this study, we developed an HALR system in a hospital to automatically and actively report pathogen test results and relevant clinical information to the TCDC ALR system. The study results show that the HALR system can improve the timeliness, sensitivity, and specificity of reported cases. Furthermore, it can provide the flexibility to integrate frequent changes to the definitions of notifiable cases when the TCDC finds new, emerging threats or diseases.

The improvement of the sensitivity and specificity of reportable cases depends not only on timely and accurate laboratory test results but also on the availability of clinical information that may be required for identified cases. This study recommends the following feasible solutions: integrate automatic pathogen reporting by the HALR system with related medical data in patients’ EMRs through the automatic system determination, with EMR data provided according to the TCDC clinical standards specifications for infectious diseases (eg, according to a “Dengue Fever” diagnosis, clinical data can include a patient’s fever [>38°C] and at least one of the following symptoms: retro-orbital pain, myalgia, arthralgia, rash, leukopenia, and hemorrhagic manifestations.)

## Abbreviations

ADRP: Automated Detection of Reportable Pathogens
ALR: Automated Laboratory Reporting
CPOE: computerized physician order entry
ELR: electronic laboratory reporting
EMR: electronic medical record
FN: false negative
FP: false positive
GBS: Group B Streptococcus
HALR: Hospital Infectious Disease Laboratory Autoreporting
ICD-9-CM: The International Classification of Diseases, Ninth Revision, Clinical Modification
LIS: Laboratory Information System
LOINC: Logical Observation Identifiers Names and Codes
NCR: Notifiable Case Reporting
NPDB: Notifiable Pathogen Database
RPU: Reportable Pathogens Update
RTCDB: Reportable Test Case Database
TCDC: Taiwan’s Centers of Disease Control
TN: true negative
TP: true positive
WebNDR: Web-based Notifiable Disease Reporting
